# Clinical outcomes of pediatric burn injuries: A Single Center Study

**DOI:** 10.12669/pjms.42.7.16510

**Published:** 2026-07

**Authors:** Hadiya Maqsood, Bushra Akram Mughal, Behram Abbas, Iqra Maqsood

**Affiliations:** 1 Hadiya Maqsood, MBBS, MRCSEd, Allied Burn and Reconstructive Surgery Center, Faisalabad Medical University, Faisalabad, Pakistan; 2 Bushra Akram Mughal, FCPS (Plast), Allied Burn and Reconstructive Surgery Center, Faisalabad Medical University, Faisalabad, Pakistan; 3 M Behram Abbas, MBBS, Allied Burn and Reconstructive Surgery Center, Faisalabad Medical University, Faisalabad, Pakistan; 4 Iqra Maqsood, MBBS, Allied Burn and Reconstructive Surgery Center, Faisalabad Medical University, Faisalabad, Pakistan

**Keywords:** Burns, burn centers, Child mortality, DALY, Debridement, Grafting, Infection, Pediatric burn, Wound healing

## Abstract

**Background and Objective:**

Burn is the leading cause of mortality and disability adjusted life years worldwide, where majority occur in low- and middle- income countries. Children under five years are particularly susceptible due to household accidents. Despite the establishment of a global burn registry, there are significant discrepancies in reported data of burn. Our aim was to evaluate the clinical outcomes of pediatric burn injuries, assess the frequency of healing and its correlation with demographic and clinical variables.

**Methodology:**

It is an observational, descriptive, longitudinal study, conducted in Allied Burn and Reconstructive Surgery Center, Faisalabad from September 2025 to December 2025. Data was collected prospectively via specialized proforma, and statistical analysis was done using SPSS 25.

**Results:**

One hundred and four patients reported with male predominance (54.8%). Mean age of presentation was 4.16 years with standard deviation of 3.01 years (range two months to 12 years). Majority lie under five years of age (74.5%). Scald burn was the most common etiology (80.8%). Mean total body surface area (TBSA%) was 16.13%. Out of 104 patients, 74 patients (71.1%) achieved healing successfully. 53.8%(n=56) healed conservatively while 17.3%(n=18) needed surgical intervention. Mechanism of burn demonstrated significant correlation with healing pattern as scald burn had greater propensity of healing conservatively (82.2%) while majority of flame (57.2%) and electric burn (75%) required surgical intervention for healing.

**Conclusion:**

Majority of burns happen due to scald incidents. However; it has shown greater healing potential and better overall outcomes as compared to other types of burn. Early assessment and diagnosis of mechanism and severity of burn can help in timely decision-making regarding better suited treatment options which is essential to improve healing outcomes in pediatric burn.

## INTRODUCTION

Burn injuries are reported to be the 5^th^ most common cause of non-fatal injuries worldwide^1^, causing a significant social, psychological, and economic impact on a child’s life along with debilitating long-term sequelae.^2^-^4^ According to WHO, around 11 million burn injuries occur globally resulting in approximately 180,000 deaths, making it one of the leading causes of mortality and disability adjusted life years worldwide.^1^ Almost half of the population contracting these injuries belongs to the pediatric age group, however, pediatric population is much less reported from low income countries.^5^ In many high-income countries, burn death rates have been decreasing, however, the rate of child deaths from burns is currently over seven times higher in low- and middle-income countries.^1^

According to WHO burn registry, global trends in pediatric burn shows that the most affected age group is from one to five years of age, with a male predominance. Almost 80-90% of such incidents occur at home due to preventable cause.^1^ Multiple strategies have been employed to ascertain the epidemiology, etiology and outcomes to curate effective preventive and standardized treatment regimes. Despite most of the burn occurring in low- and middle- income countries, the data is vastly underreported. Global Burn Registry (GBR) was established in 2018 to enhance the understanding of burn injuries, identify prevention strategies, and set benchmarks for acute burn care. It still fails to represent comprehensive picture of global trends because of multiple factors including voluntary involvement in the registry, small number of countries contributing to the data, and lack of pediatric specific care facilities to name the few.^5^

Developed countries have specialized burn centers, comprehensive registries, and effective awareness programs, whereas many low-resource regions lack systematic data and pediatric burn care services, contributing to both underreporting and poor clinical outcomes.^5^,^6^ Despite the significant disease burden, no designated pediatric burn center facility is available in the county. Moreover, there are notable information gaps in our region mainly due to beforementioned reasons. The aim of this study was to analyze sequelae of pediatric burn injuries. This will help generate local data to describe burden and its impact on life post recovery, device systemic awareness programs, develop and enforce effective policies in pediatric burn care.

## METHODOLOGY

This observational, descriptive longitudinal study was conducted in Allied Burn and Reconstructive Surgery Center, Faisalabad from September 2025 to December 2025. In this study all patients, both genders, who presented to our center and met the inclusion criteria were added in the study via non-probability, consecutive sampling technique.

### Inclusion and Exclusion criteria:

All the patients, both male and female, from the 1st day of life till 12 years of age, presenting to our center with TBSA up to 40% of mixed thickness burn, contracted within last seven days were included in the study. Deep thickness Burn of >40%, delayed presentation of more than seven days and those presenting with post burn complications e.g., keloids, hypertrophic scars were excluded.

### Ethical approval:

Approval from the ethical review board of Faisalabad Medical University was obtained. (Ref. No.48ERC/FMU/2024-25/107; dated July 28, 2025).

### Treatment protocol:

Data was collected prospectively through special Performa by detailed history and clinical examination. All patients were received in hospital’s emergency department where primary and secondary survey was done according to ATLS protocols. After that, patients requiring specialized burn care according to international guidelines (children with >10% TBSA, burn involving critical areas including head & neck, hands, perineum, all cases of electric burn, suspicion of child abuse), were referred to our center. Diagnosis of burn was made by calculating size and depth of burn. Burn size was calculated in terms of total burn surface area (%) by Lund and Browder chart. Burn depth was determined in terms of the degree of burn by the examination of clinical signs. Burn with hyperemia, blanching, detached epidermis with unexposed dermis was characterized as superficial thickness burn. Blisters, Exposed dermis with shiny moist pink surface, blanching and extremely painful was categorized as partial thickness burn. White, leathery, dry, non -blanching and painless burn was considered deep thickness burn.^7^ However, the majority of the patients showed mixed thickness characteristics as burn is a dynamic condition in first 48-72 hours which evolves on the basis of first aid and initial resuscitation. Hence, labeled as mixed thickness burn in acute phase (first 48-72 hours).^7^ All patients got treated with general principles of burn management i.e., fluid resuscitation with urine output monitoring, early and effective analgesia, burn wound wash and aseptic moist protective dressing, infection control, blood and its products transfusion, physiotherapy, psychological, and nutritional support and surgical intervention where indicated.

During the acute phase of burn, patients were closely monitored for limb- and life- threatening conditions and interventions were done accordingly. Fasciotomy and escharotomy was done for compartment syndrome, tracheotomy for laryngeal edema in case of inhalational injury, amputation for mummified limb in case of electrical injury. Dressing (petroleum-impregnated gauze dressing (Para tulle, Bactigras)) was done after proper wound wash and was changed on alternate days under aseptic environment. The wound was assessed clinically for improvement. Presence of skin appendages and decrease in size of burn wound with presence of new pink epithelial tissue were considered positive signs of improvement. Patients were kept in an in-patient facility until hemodynamic stability was achieved and were discharged once stable and improving with proper guidance of dietary care, dressing protocols and emergency signs of return to hospital. They were followed up in out-patient department (OPD) every 3^rd^ day until complete healing was achieved.

Wound was assessed by two consultant plastic surgeons independently, double blinded by the process on 21st day after burn for complete healing. As superficial and partial thickness burn with potential of primary healing generally heals by week two to three.^7^ Presence of new epithelial tissue over wound surface and formation of new epithelial lip over wound edges was labeled as healed without surgical intervention. Decision for surgery was made on clinical characteristics of burn wound after 48 to 72 hours. Wounds which showed characteristics of deep thickness burn underwent early surgical excision and grafting. Moreover, wounds which failed to show improvement with signs of epithelialization on serial dressings and wounds which got complicated and demarcated into deep thickness due to infection, dehydration, anemia also underwent surgical debridement and grafting. After successful take of graft, these patients were labelled as healed with surgical intervention.

### Outcome measures:

The primary outcome was frequency of healing at 21 days after initial burn injury, categorized as:

- Healed without surgical intervention.
- Healed with surgical intervention.
- No healing.

### Statistical analysis:

The collected data was entered and analyzed using Statistical Package for Social Sciences (SPSS) version 25. Quantitative variables including age and TBSA were represented as Mean ± S.D. Qualitative variable including gender, mechanism of burn, outcomes were represented as frequencies and percentages. Chi-square test was applied to assess association between healing outcome and independent variables. A p-value < 0.05 was considered statistically significant.

### Sample size:

The sample was calculated as follows:

- P = 60.63% (30% healed without complications + 30.6% healed with complications).
- Confidence level = 95%,
- Absolute precision = 10%.

The minimum required sample size was 92; however, 104 patients were enrolled during the study period (September–December 2025) using non-probability consecutive sampling.

## RESULTS

One hundred and four pediatric burn patients presented, males (54.8%; n = 57) slightly outnumbered females (45.2%; n = 47). Mean age of presentation was 4.16 years with standard deviation of 3.01 years (range 2 months to 12 years). Mean total body surface area (TBSA) was 16.13 with standard deviation (SD) of 9.68. More than two-third of patients were under 5 years of age (74%; n=77). Scald burns were the most common mode of injury (80.8%; n=84), followed by flame and electrical burns.

Overall, 74 patients (71%) achieved complete healing by day 21. Among them, 56 (53.8%) healed without surgical intervention and 18 (17.3%) required surgery. 30 (29%) patients were not healed amongst whom twenty-one patients (20.2%) left against medical advice (LAMA) before completion of treatment, and nine patients (8.7%) expired.

Significant association between mechanism of burn and healing outcomes was noticed with chi-square test of independence X^2^ (1, 104) = 11.029, p-value 0.004 (<0.01), Ф = 0.389. Patients with scald injuries (82.2%; n=51) were more frequently managed conservatively. While patients with flame (57%; n=4) and electrical burns (75%; n=3) had a higher likelihood of requiring surgical intervention. The majority of the surgical intervention needed were debridement (45.5%; n=10) and grafting (81.9%; n=18) for deep thickness burns and burns complicated by wound infections. Other interventions included fasciotomies (9.1%; n=2) for compartment syndrome, amputations (18.2%; n=4) for electric burn injuries. However, the value of phi-coefficient was 0.389 which indicates small to moderate effect size. This suggests that differences across mechanism of burns were present, but the strength of the relationship was relatively modest, indicating only a limited proportion of the variation in responses.

Both male and female patients showed higher frequency of healing without surgical intervention. However, greater number of female patients (36.3%) underwent surgical interventions as compared to males (15%). There was statistically significant association noticed between gender and healing outcomes X^2^ (1, 104) = 4.442, p-value 0.035 (< 0.05), Ф = 0.247. The value of phi-coefficient is 0.247, indicating a small association between gender and healing outcome. This finding suggests that gender differences were present, however, the strength of the relationship was relatively weak, indicating that gender accounted for only a limited proportion of the variation in responses.

Age failed to show significant association with healing X^2^ (1, 104) = 3.34, p-value 0.342 (>0.05), Ф = 0.214. The p-value is >0.05 indicating no significant association. However, Infants, toddlers, and children showed higher frequency of healing without surgical intervention, while adolescents showed higher frequency of healing with surgical intervention. This can be attributed to increase in incidence of flame and electric burn with increasing age.

## DISCUSSION

This study was designed to assess the frequency of healing in pediatric burn population. Our study reinforces that scald burn is the leading cause of pediatric burn injuries. Most commonly affected population lies under five years (Table-I) likely due to household incidents, caregiver inattention, underdeveloped motor skills. Moreover, the morphological differences including thin skin, decreased body surface area, and lack of an established immune system make them prone to severe injuries caused by similar insults as compared to adult.^8^ This aligns with the observation of Özlü Ö et al that majority of preschoolers suffer from burn injuries due to spillage of hot liquids at home.^9^ This emphasizes on the preventable nature of these incidents which can be reduced with effective educational programs and preventive policies. A recent narrative review suggested that children from one to three years exhibit the highest burn incidence rate.^8^ Avci et al. reported males between age two to four years are the most susceptible.^10^ There was slight male predominance (Table-I), however, similar healing patterns were noticed in both genders. Age failed to show a statistically significant correlation with the healing potential while gender showed minor association, suggesting that they might not be powerful predictors of short-term healing.

**Table-I:** Demographic variables of Study (N = 104).

Variables	f	%
***Gender***		
Male	57	54.8
Female	47	45.2
***Age***		
Infants (<1 year)	9	8.7
Toddlers (1-5 years)	68	65.4
Children (6-10 years)	21	20.2
Adolescents (>10 years)	6	5.8
***Mechanism of Burn***		
Flame	16	15.4
Scald	84	80.8
Electric	4	3.8
***Outcomes***		
***Healing***	74	71.1
Without Surgical Intervention	56	53.8
With Surgical Intervention	18	17.3
***No healing***	30	29
LAMA	21	20.2
Expire	9	8.7

***Note:*** F= Frequency, %= Percentage.

The frequency of healing by 21^st^ day was 71.1% with most of the patients recovering without surgical intervention. These statistics exceed earlier average from our region where Anwar et al reported healing rate of 60.63%^11^ indicating improved acute care delivery. The mortality rate was 8.7% which is significantly lower than the previous national data where mean mortality rate was reported to be 14.84%.^12^ It can be due to early hospital presentation, prompt treatment, careful monitoring and early decision-making for surgery. However, patients with limited healing potential (deep thickness burns >40% and delayed presentations of >7 days) were excluded which might have affected the estimation of overall mortality.

The mechanism of injury emerged as the most significant predictor of the healing pathway (p < 0.01). Scald burn- the most common etiology- showed greater propensity of healing with conservative management while flame and electric burn required surgical intervention more (Table-II). The given observation is clinically plausible because flame and electrical burns tend to cause more severe tissue damage as demonstrated in previous studies^13^-^15^, predisposing the patients to increased risk of complications like delayed wound healing, prolonged hypermetabolic state, rapid depletion of blood reserves and anemia, wound infections leading to sepsis and septic shock causing death.

**Table-II:** Frequencies and Chi-Square results for Healing with and without intervention (N=104).

	Total patients	Healing without surgical intervention		Healing with surgical intervention			
		n	%	n	%	X^2^	P-value
***Age***						3.34	0.342*
Infants (<1 year)	9	3	75	1	25		
Toddlers (1-5 years)	68	39	78	11	22		
Children (6-10 years)	21	13	72	5	28		
Adolescents (>10 years)	6	0	0	1	100		
***Gender***						4.442	0.035**
Male	57	34	85	6	15		
Female	47	21	63.6	12	36.3		
***Mechanism***						11.029	0.004**
Scald	84	51	82.2	11	17.7		
Flame	16	3	42.8	4	57.2		
Electric	4	1	25	3	75		

* p>0.05

** p<0.05.

**Table-III:** Frequencies and Percentage of outcome (N=104).

Variables	Category	f	%	Total cases
***Healing***				
Yes	Without Surgical Intervention	56	53.8	74
	With Surgical Intervention	18	17.3	
No	LAMA	21	20.2	30
	Expire	9	8.7	

While there was no statistically significant association of gender with final healing outcomes, increased surgical burden was noticed in female population as they were nearly twice as likely to undergo surgical intervention for healing. As reported in previous study, this finding can be attributed to more severe burn injuries contracted by females due to household and cooking related incidents.^16^ A recent study on global burden of burn injuries showed that females consistently exhibited higher years lived with disability (YLDs) due to severe burns, a pattern which persisted in all age groups.^17^ Contrarily, another study from Romania reported that males are likely to suffer from more severe flame burn injuries.^14^ This difference reflects different socio-cultural responsibilities and exposure patterns in different parts of the world. Correlation of age with surgical intervention showed that a greater proportion of patients underwent surgical interventions with increasing age.^6^,^18^ This finding is in coherence with existing literature where adolescents are more likely to suffer from flame and electric burns.^8^,^13^ Most common surgical intervention needed was grafting followed by debridement, amputations in case of electric burn and fasciotomies for compartment syndrome as demonstrated in the previous studies.^19^

While the healing outcomes show promising results comparable with previous regional, national and international studies (Table-II), there was a significant proportion of patients who left against medical advice (LAMA), which could have impacted outcome assessment and demonstrates continuity of care issues. This has previously proven to be the cause of discrepancies in data reported in the global burn registry.^5^,^6^,^17^

Our study is the first of its kind from our region which has prospectively studied pediatric burn injuries, correlated healing outcomes with demographic (age, gender) and clinical variables (mechanism of burn), and compared the need for surgical intervention and factors influencing it, bridging the gap between local data regarding pediatric burn injuries. Clinically, these findings underscore the need to establish the severity and mechanism of burn early in order to help make appropriate decisions about the management of burns. Although conservative management is successful with the majority of scald injuries, flame and electrical burns should be observed more and assessed for surgery at the earliest opportunity to achieve the best results. The increased incidence of these injuries in children under five years of age with the majority of them occurring in household settings reinforces the importance of effective preventive strategies, awareness campaigns and policy making on government level.

**Fig.1:**
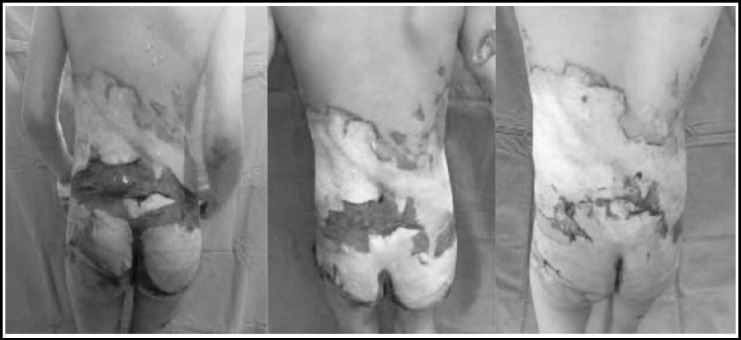
Shows mixed thickness scald burn healed by conservative management (A) Post-burn day 0 (B) Post-burn day 9 (C) Post-burn day 15.

**Fig.2:**
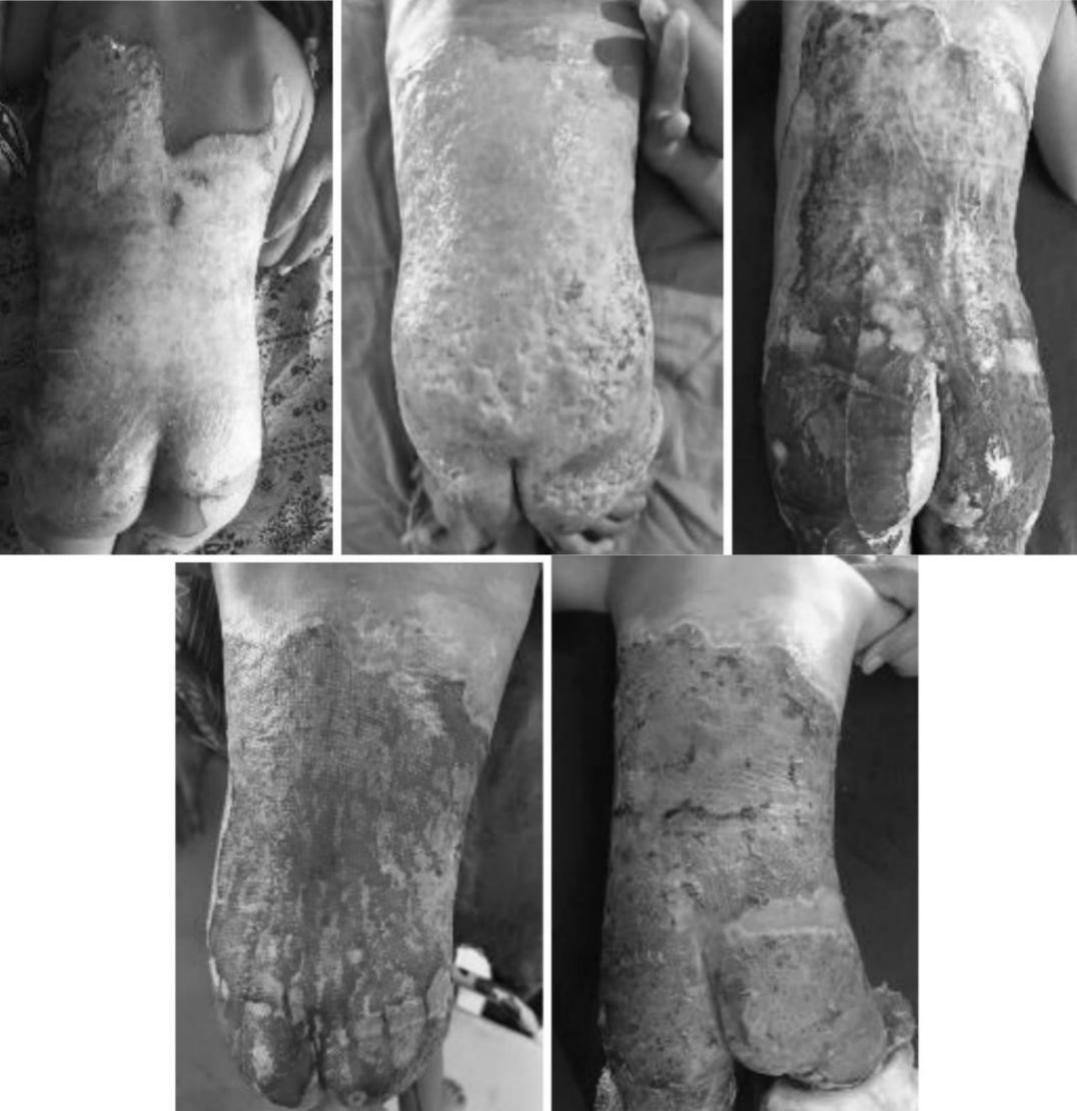
shows deep thickness flame burn healed with surgical intervention (A) initial presentation (B) after 1^st^ debridement (C) after 2^nd^ debridement (D) after 3^rd^ debridement (E) secondary wound coverage with split thickness skin grafting.

### Limitations:

This study was conducted in only one institution with a small sample size of 104 to report the short-term clinical outcome of pediatric burn, so these findings cannot be generalized to a larger population and for that long duration, multi-center studies with larger sample sizes are needed. The healing rate was evaluated 21 days after initial burn injury. Long-term sequelae including functional or aesthetic outcomes, complications like scarring and contractures were not studied to ascertain the impact of these injuries on morbidity and quality of life. In addition, the patients leaving against medical advice (LAMA) discontinuing treatment could have created bias on the outcome reporting.

## CONCLUSIONS

Pediatric burn injuries pose a significant health care challenge in the developing world demonstrating varied healing outcomes depending on the mechanism and severity of injury. Majority of burn in this age group happen due to preventable scald incidents and most susceptible age group is under five years of age. However, scald burn has greater propensity of healing with conservative management, showing greater healing potential while surgical intervention remains crucial for selected mode of injuries including flame and electric burns reflecting increased severity of injury. Early assessment, prompt treatment and timely surgical decision-making are essential to improve healing outcomes in pediatric burns. Moreover, focusing on prevention and first-aid, standardizing burn care facilities, and establishment of specialized pediatric burn centers with all essential facilities according to the vulnerable age group can improve outcomes.

### Authors’ Contribution:

**HM:** Conceived the idea, designed the study, and did statistical analysis & editing of manuscript, is responsible for integrity of research.

**HM, MBA & IM:** Did data collection, critical review and manuscript writing.

**BAM:** Designed the study and did review and final approval of manuscript.
